# Patients’ time perception in the waiting room of an ambulatory emergency unit: a cross-sectional study

**DOI:** 10.1186/s12873-019-0254-1

**Published:** 2019-08-01

**Authors:** Hervé Spechbach, Jessica Rochat, Jean-Michel Gaspoz, Christian Lovis, Frederic Ehrler

**Affiliations:** 1 Division of Primary Care Medicine, Department of Community Medicine, Primary Care and Emergency Medicine, Geneva University Hospitals, Geneva, Switzerland; 2 Faculty of Medicine, University of Geneva, Geneva, Switzerland; 3 Division of Medical Information Sciences, Geneva University Hospitals, Geneva, Switzerland

**Keywords:** Patient satisfaction, Emergency department, Waiting time, Perceived waiting time, Service-oriented health care

## Abstract

**Background:**

Patient satisfaction has become an increasingly important element in a service-oriented healthcare market. Although satisfaction is influenced by many factors, the waiting time to be seen by medical staff has been shown to be one of the key criteria. However, waiting is not an objective experience and several factors can influence its perception.

**Methods:**

We conducted a questionnaire-based, cross-sectional study among patients attending the emergency unit of a Swiss university hospital in order to explore the key factors influencing wait perception.

**Results:**

A total of 509 patients participated in the study. Appropriate assessment of emergency level by caregivers, the feeling of being forgotten, respect of privacy, and lack of information on the exact waiting time were identified as significant variables for wait perception.

**Conclusions:**

Our study confirmed the existence of a ‘golden hour’ when the patient is willing to wait until the medical encounter. In case the wait cannot be limited, an appropriate assessment of the emergency level by caregivers and avoiding the patients of feeling being forgotten are very important factors to avoid a negative perception of the waiting time before seeing a doctor.

**Trial registration:**

(ID REQ-2016-00555).

## Background

The emergency department (ED) of a health facility provides services whose evaluation is strongly linked to the ‘customer’ experience [1]. In a service-oriented healthcare market, patient satisfaction is a quality indicator receiving increasing attention. Dissatisfied patients are likely to share their negative experience with other people, thus having a negative influence on service perception [2, 3]. In addition, internet promotes a rapid and wide dissemination of these opinions. This word-of-mouth marketing is powerful, especially as consumers grow more knowledgeable about their healthcare choices. More importantly, there is evidence of a reciprocal relationship between patient satisfaction and continuity of care, which is associated with improved patient outcomes. For instance, satisfaction with care has been shown to have a significant influence on patient adherence with treatment plans [4].

It is difficult to explore the holistic experience of being a patient as consideration must be given to the multiple facets of his/her psychological behaviour related to the cultural aspect of care [5]. Waiting time is considered to be an important determinant of patient satisfaction and should be carefully considered. Bursch et al. [6] showed that the duration of waiting time before ED care, patients’ evaluation of care by doctors and nurses, the organization of ED staff, and quality of received information were the most important variables associated with an overall satisfaction with ED services. A Swedish study [7] including 758 patients investigated patient satisfaction with treatment and service at an ED. Results showed that satisfaction was lower among patients who were triaged as non-urgent (and thus waited longer) than among the immediate and urgent triaged patients [8]. In the USA, the Kaiser Permanente’s integrated health care model [9] identified that the most important variable associated with overall satisfaction with ED services was satisfaction with the amount of time spent waiting before the patient was cared for in the ED.

If the actual waiting time is a strong factor for satisfaction, its perception is also a key factor. Marketing and consumer research literature [10] found that satisfaction is mainly influenced by the difference between the consumers’ acceptable and perceived waiting time, with a linear relation between actual and perceived waiting time [11, 12]. Similarly, several international studies conducted in the ED showed that satisfaction is strongly correlated with perceived waiting time and its correlation with the actual waiting time. Waiting time at the ED is often perceived as unreasonably long and information received from nurses and doctors is judged to be unsatisfactory [13, 14]. Among the factors influencing the perceived waiting time, the role of the expected waiting time can be highlighted. The perceived waiting time influences the satisfaction we have from a service through our expectation (i.e. the expected waiting time) and is explained by the disconfirmation paradigm perceptions of a service [15] in that dissatisfaction arises when service expectations are not met (i.e. a positive disconfirmation occurs when a service is perceived as being better than expected and a negative disconfirmation occurs when a service is perceived as being worse than expected). Growing pressure to provide care for more patients over the last decade has led to overcrowding and longer waiting times [16], thus leading to dissatisfaction from ED patients. Moreover, it has been demonstrated that the longer a patient waits before receiving care, the more s/he is at risk of leaving without being seen [17]. In this context, it is critical to understand clearly the factors influencing the perception of wait of ED patients.

In Switzerland, a consultation at the ED of a public hospital results in a charge of several hundred francs to patients who pay one part, the other part being reimbursed by their health insurance. Therefore, patients expect a certain level of service and may complain when dissatisfied (0.3% of visits). Among patient complaints, those concerning the waiting time until a medical contact are recurrent at Geneva University Hospitals, although not frequent (0.1% of visits), similar to other centres elsewhere [18–20]. As a consequence, the hospital management has committed itself to improving the situation and thus enhance the institutional image among the local population. In this context, our study aims to investigate the different factors influencing the perception of waiting time until medical contact through a questionnaire distributed to patients triaged as non-urgent in the outpatient emergency unit. We did not include other parameters of quality of care in our analysis, such as the actual medical care or appropriate diagnosis and follow-up, and focused only on the emergency unit triage and the initial patient throughput until a medical contact.

## Methods

### Study design

We conducted a questionnaire-based, cross-sectional, descriptive study between 17 December 2015 and 14 March 2016.

### Setting

The survey was conducted at the outpatient emergency unit of Geneva University Hospitals, the largest university hospital in Switzerland with over 1900 beds. The unit is separated into ‘orange’ and ‘green’ subunits. The ‘orange’ subunit cares for general ambulatory pathologies and the ‘green’ subunit for surgical ambulatory pathologies. Both have six consultation rooms each. Each subunit has its own semi-closed waiting room with seating, a television, water and newspapers. The staff (doctors and nurses) are the same for the entire unit and work part of the week in the ‘orange’ subunit and another part in the ‘green’ subunit. Both units open at 8 h00 and close at 23 h00. After 21 h00, if more than six patients are already being cared for and a further six are in the waiting room, the units are considered as closed and all patients arriving after that time remain in the inpatient emergency department, which is open 24 h/24 h. At 23 h00 all patients present in the outpatient emergency unit are transferred to the inpatient unit. In 2016, 22,000 patients consulted the unit (half in the ‘orange’ and half in the ‘green’). Median length of stay is 3 h in the ‘orange’ subunit and 2.8 h in the ‘green’ subunit.

When a patient arrives at the emergency entrance, s/he is first seen by a triage nurse who decides if the patient is a candidate for the ambulatory emergency unit, based on the Swiss Emergency Triage Scale (SETS). Level 1 is a life−/limb-threatening situation where the patient must be seen by a medical doctor immediately, level 2 in the following 20 min, level 3 in 120 min, and level 4 is considered as non-urgent. Eighty percent of patients visiting the emergency unit are classified as level 3 and 10% as level 4. After triage, the patient goes through an administrative registration process and is then directed to one of the subunits by following coloured lines on the floor. These lead to a nursing desk where a nurse accompanies the patient to the waiting area. Both subunits have their own semi-closed waiting room. Whenever possible, nurses inform patients about the waiting time using their own estimation. As soon as a consultation room and a doctor are available, the patient is taken to the room by the nurse. After the medical consultation, the patient can either return home or may have to undergo an additional examination and return again to the waiting room. A small percentage of patients (5%) are hospitalized and 5% leave the unit without being seen by a doctor.

### Population

**Fig. 1:**
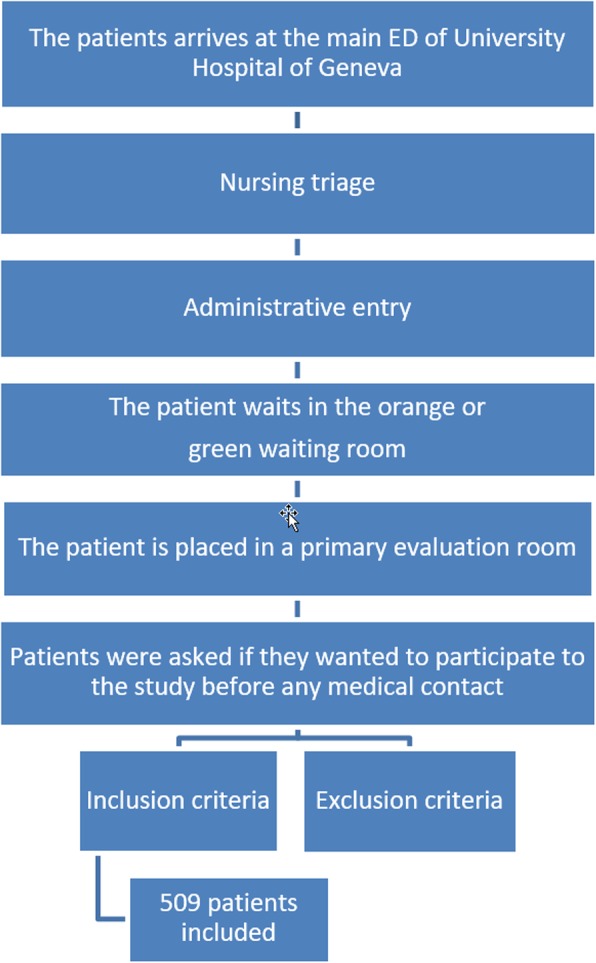
Study flow chart

Patients presenting to the outpatient emergency unit were invited to participate to the study if they were at least 16 years old and French-speaking. Exclusion criteria were patients not capable of discernment (e.g. unconscious, under the influence of drugs, suffering extreme trauma or with cognitive disorders), unable to read/understand French, vision problems, those who had already completed the questionnaire, and patients in severe pain or too aggressive. The patient journey until data collection is shown in Fig. 1.

### Questionnaire

The questionnaire developed for this study was based on the most frequent factors related to waiting time perception and overall satisfaction in the ED identified in the literature [21–29] regarding expected waiting time: the patient’s estimate of the maximum reasonable waiting time before seeing a doctor; perceived waiting time; appropriate assessment of emergency level by caregivers; perception of their own emergency level compared to other patients; satisfaction with information received; organization of the emergency unit; feeling of being forgotten; frustration; capability to position themselves in the waiting queue; respect of privacy; comfort in the waiting room; and an indication of waiting time by the nurse. Additional items were added to explore specific topics of interest, such as the recommendation of the emergency unit to others, the interest in waiting elsewhere and overall satisfaction. The final questionnaire consisted of 27 items. Most items were evaluated with a 5-point Likert scale ranging from ‘strongly disagree’ to ‘strongly agree’. The item concerning the perceived waiting time was evaluated with a 5-point Likert scale ranging from ‘very short’ to ‘very long’. The item concerning the perception of their own emergency level compared to other patients was evaluated with a 3-point Likert scale ranging from ‘superior’ to ‘inferior’. The two items concerning the expected waiting time and the patient’s estimate of the maximum waiting time before seeing a doctor were questions requiring a time input in hours and minutes. To collect the real waiting time, we extracted the time that the patient actually saw the doctor from the clinical information system (CIS). Indeed, each time a doctor starts the medical care, s/he notifies the CIS by clicking a specific button and thus generates a log into the system

### Procedure and ethical considerations

The institutional ethics committee approved the study protocol. Participation to the study was voluntary and by oral consent. Questionnaires were distributed over a period of three months by a total of 20 nurses during two different shifts: 7 h30 to 16 h00 and 15 h00 to 23 h30.

Following a convenience sampling approach, each questionnaire was distributed to a patient by a nurse in a consultation room while waiting for the doctor. We did not include patients who did not wait before going to a consultation room. Following verification of the inclusion criteria, the nurse asked the patient if s/he agreed to complete the questionnaire. Information about the study and confidentiality were given orally. The survey took approximately 20 min to complete. The nurse remained available for any questions and to help the patient complete the questionnaire if necessary. Once completed, questionnaires were collected by the nurses and placed in a dedicated box in a secure room. Questionnaires were collected by a scientific collaborator each morning and entered into an Excel file. In order to link the questionnaire data to the data extracted from the CIS, we used a mapping file linking the ID of the questionnaire to the patient ID. Once all data were included in the Excel file, only the questionnaire ID was retained to ensure anonymous analysis.

### Statistical analysis

Data were analyzed using Stata/IC 14 software [30]. Descriptive statistics and frequencies were produced to describe the demographic and medical characteristics of participants. Variables evaluated with a 5-point Likert scale were re-coded into three categories: ‘disagree’, ‘neither disagree nor agree’, and ‘agree’. In addition, the actual waiting time of each patient that responded to the questionnaire was extracted from the hospital CIS. The variable ‘difference between actual and expected time’ was created by subtracting the duration that the patient thought that s/he should have waited before being seen by a doctor from the actual duration of the patient wait. If the value was superior to zero, it meant that the patient waited longer than expected. Statistically significant predictors of the waiting time perception were determined by an ordered logistic regression using every variable as input. Missing data were removed from the regression analysis.

## Results

A total of 509 patients participated in the study. The demographic characteristics of participants are presented in Table 1.

**Table 1:** Demographic characteristics of respondents

Demographic characteristics	Respondents
	Mean (standard deviation)
Age (years)	42 (17)
	n (%)
Sex	
Male	266 (52.3)
Female	243 (47.7)
Nationality	
Swiss	268 (52.7)
French	63 (12.4)
Portuguese	44 (8.6)
Spanish	17 (3.3)
Italian	15 (2.9)
Others	102 (20.1)

Table 2 shows the medical characteristics of respondents. Most respondents (88.5%) were classified with an emergency level of 3, 3.5% were level 2, and 8% were triaged as level 4 (the less urgent).

**Table 2:** Medical characteristics of respondents

Medical characteristics	Respondents
	n (%)
Emergency level according to SETS	
1	0 (0)
2	18 (3.5)
3	450 (88.4)
4	41 (8.1)
Reason for ED visit	
Limb trauma	128 (25.1)
Skin/soft tissue ailment/infection	54 (10.6)
Arthralgia, myalgia, neuralgia	45 (8.8)
Pain and/or oedema of a limb	36 (7.1)
Abdominal pain	24 (4.7)
Cervicalgia, back pain, low back pain	24 (4.7)
Influenza syndrome	24 (4.7)
Superficial wounds	17 (3.3)
Cough, sputum	15 (2.9)
Others	142 (28.1)
Family doctor	
Yes	379 (74.5)
No	127 (25)
Missing data	3 (0.5)
Already consulted at the same emergency unit	
Yes	355 (69.7)
No	154 (30.3)

*SETS* Swiss Emergency Triage Scale

### Relation between wait perception and exact waiting time

**Fig. 2:**
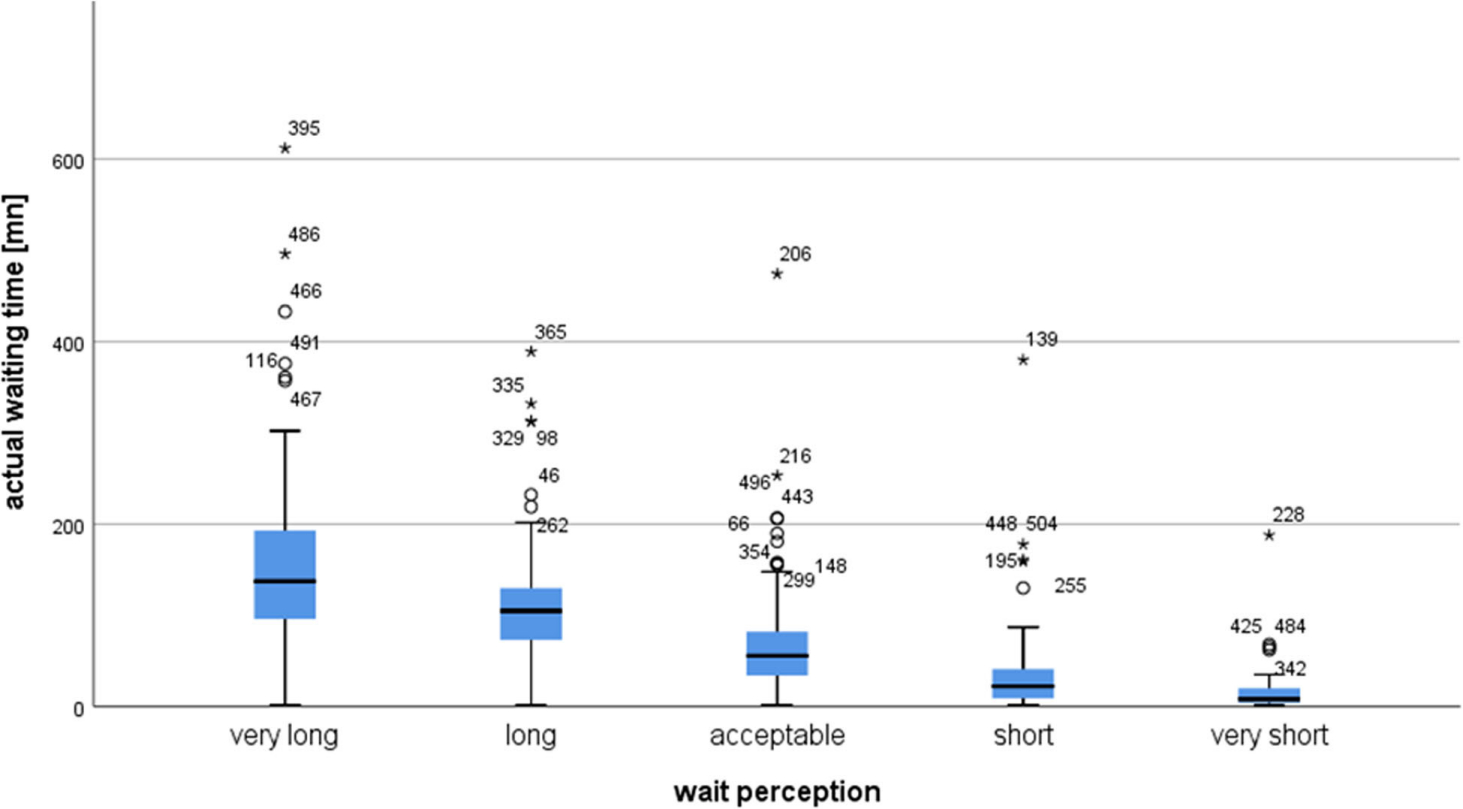
Average waiting time according to wait perception

Figure 2 illustrates the wait perception according to the actual waiting time and suggests that the perception of waiting time is directly correlated to its duration. Patients perceiving the wait as very long were those who waited the most on average. A non-parametric ANOVA (Kruskal-Wallis test) was performed to verify if the mean differences of the actual waiting time according to the five modalities of the wait perception (i.e. very short, short, acceptable, long, very long) were statistically significant. The non-parametric test showed a statistically significant difference (Chi-square = 220; *p* < 0.001). To define which differences were significant, 10 multiple comparisons were performed using the Mann-Whitney test, taking into account the Bonferroni correction. All differences were statistically significant with a *p*-value inferior to 0.001. This result proves that the actual waiting time influences significantly the perception of the wait. Figure 2 shows that wait perception is considered as very short, short and acceptable under an exact waiting time mean of 1.03 h (standard deviation = 0.50).

### Relation between wait perception and wait confirmation

**Fig. 3:**
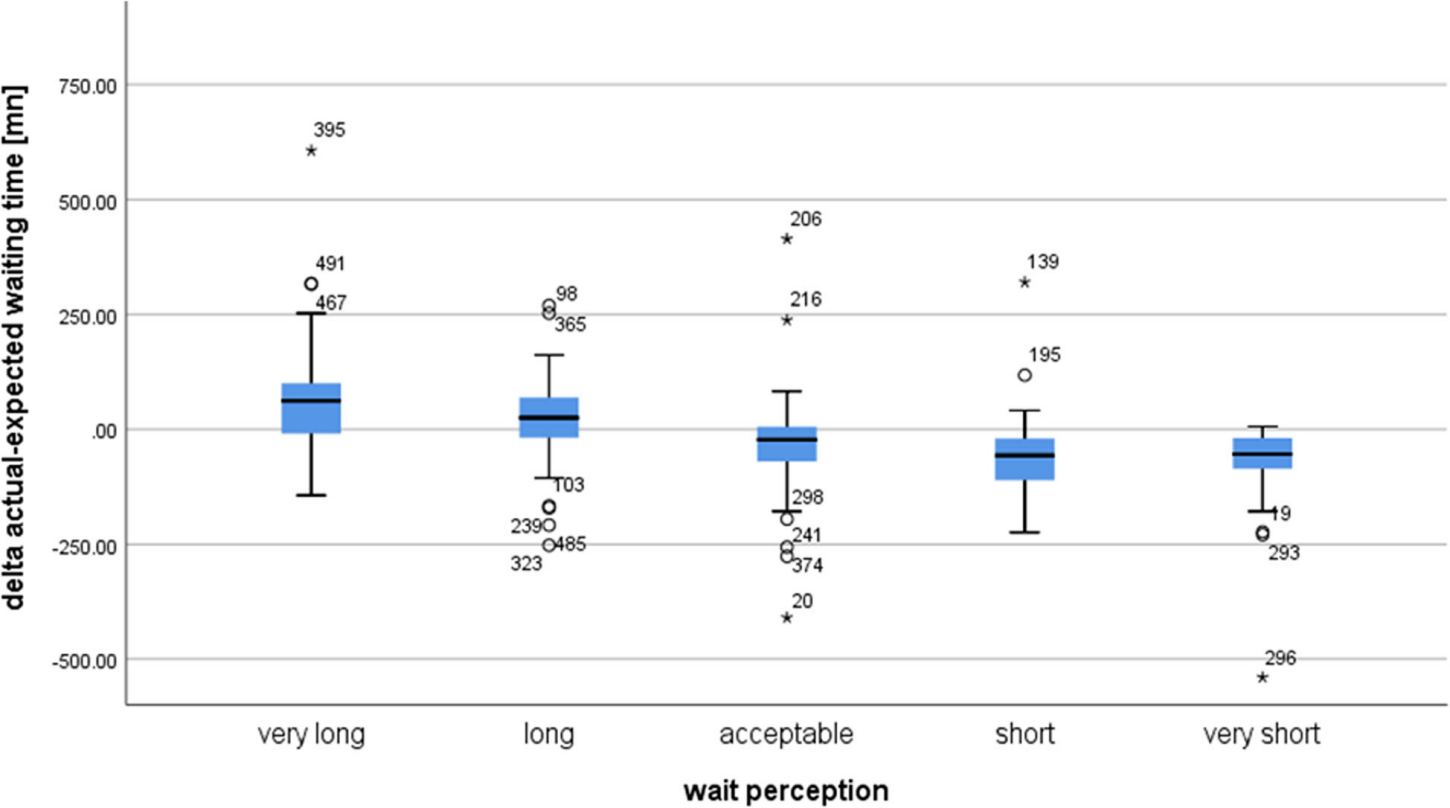
Average difference between the actual and expected waiting time according to the wait perception

The difference between the actual and expected waiting time, known as ‘wait confirmation’, measures the difference between the expectation and the reality. Patients whose actual wait exceeded the expected waiting time perceived the wait as very long (Fig. 3). A non-parametric ANOVA (Kruskal-Wallis test) was performed to verify if the mean differences between the delta actual-expected waiting time according to the five modalities of the wait perception (very short, short, acceptable, long, very long) were statistically significant. The non-parametric test showed a statistically significant difference (Chi-square = 112; *p* < 0.001). To define which differences were significant, 10 multiple comparisons were performed using the Mann-Whitney test, taking into account the Bonferroni correction. Apart from the difference between the ‘very long’ and ‘long’ categories, all differences were statistically significant with a *p*-value inferior to 0.001. This result proves that the difference between the actual and expected time significantly influences the wait perception.

### Ordered logistic regression of the wait perception

We performed an ordered logistic regression to identify the factors influencing the wait perception (Table 3) on the 325 observations without missing data. All variables that were statistically significant in univariate analysis were integrated into the multivariate model. The reference value was ‘neither disagree nor agree’, except for the variable ‘exact waiting time’, which was continuous.

The outcome variable was the perception of the waiting time and the reference category was ‘very short’. The most influencing factor of the wait perception was the appropriate assessment of the patient emergency level by caregivers. The odds of perceiving the wait as ‘very short’ were 5.37 times (95% CI 2.46–11.73; *p* < 0.001) greater for a patient considering their emergency level adequately assessed compared to a patient answering ‘neither disagree nor agree’. The second factor was the ‘feeling of being forgotten’. In Tables 4, 23% (115/499) of respondents reported having felt forgotten. The odds of perceiving the wait as ‘very short’ were three times (95% CI 1.47–6.15; *p* < 0.001) greater for a patient that did not feel forgotten in the waiting room compared to a patient answering ‘neither disagree nor agree’. The other predictor was ‘respect of privacy’. Table 4 shows that 5.8% (29/495) reported that their privacy was not respected in the waiting room. The odds of perceiving the wait as ‘very short’ were 4.76 times lower (odds ratio [OR] = 0.21; 95% CI 0.05–0.88; *p* = 0.03) for a patient considering that his/her privacy was not respected compared to a patient answering ‘neither disagree nor agree’.

**Table 3:** Ordered logistic regression of the wait perception: the significant factors

	Univariate analysis				Multivariate analysis			
	Odds ratio	*p* > |z|	[95% CI]		Odds ratio	*p* > |z|	[95% CI]	
Perception of the waiting time								
Appropriate assessment of emergency level by caregivers								
Agree	12.07	< 0.001	5.15	28.30	5.37	< 0.001	2.46	11.73
Feeling of being forgotten								
Disagree	5.2	< 0.001	3.18	8.49	3.00	< 0.001	1.47	6.15
Respect of privacy								
Disagree	0.26	< 0.001	.11	.59	.21	0.03	0.05	0.88
Exact waiting time	0.98	< 0.001	.97	.98	.98	< 0.001	0.98	0.99

**Table 4:** Descriptive statistics

Variable	Strongly disagree (%)	Disagree (%)	Neither agree nor disagree (%)	Agree (%)	Strongly agree (%)	Missing data (N)
Satisfaction in the waiting room	4.8	6.2	17.9	41.0	30.0	12
Waiting time matches patients’ expectations	12.6	20.3	13.2	35.4	18.5	17
Appropriate assessment of emergency level by caregivers	0.8	3.7	15.4	51.0	29.1	17
Satisfaction with information received about the stages of care in the emergency unit	1.6	3.0	12.7	52.2	30.5	11
Organization of the emergency unit	2.2	6.0	16.0	52.5	23.4	8
Feeling of being forgotten	31.5	30.1	15.4	16.0	7.0	10
Frustration caused by patients being seen before you	31.6	28.1	25.9	9.1	5.2	104
Being able to position yourself in the waiting queue	10.1	12.5	29.5	34.1	13.8	52
Interested in waiting elsewhere	7.9	8.3	16.3	31.1	36.4	17
Respect of privacy	2.4	3.4	9.9	47.9	36.4	14
Discomfort disrupted by other patients	39.8	28.3	17.5	10.7	3.7	22
Discomfort disrupted by the coming and going of caregivers	45.6	30.0	16.0	5.3	3.1	22
Discomfort related to the material/furniture	38.1	28	17.5	11.3	5.1	23
Discomfort disrupted by the lack of occupations/distractions	28.5	26.8	23.5	15.7	5.6	24
Would recommend the emergency unit to others	2.0	5.4	15.9	41.3	35.3	13
	Superior (%)	Similar (%)	Inferior (%)	Missing data		
Patient perception of their own emergency level compared to other patients	9.2	68.7	22.1	42		
	Not informed (%)	Informed	Cannot remember (%)	Missing data (N)		
Indication of a waiting time by the nurse	53.2	35.8	11.0	18		
	Mean	Standard deviation	N	Missing data (N)		
Waiting time expected by the patient (min)	94.85	63.68	481	28		
Patient’s estimation of the maximum reasonable waiting time before seeing a doctor (min)	65.42	47.97	472	37		
Actual waiting time of the patient (minutes)	78	77	508	1		
	Very long (%)	Long (%)	Acceptable (%)	Short (%)	Very short (%)	Missing data (N)
Perception of the waiting time	13.1	19.3	43.5	14.1	9.9	6

The last predictor was the actual waiting time with an OR of 0.98 (95% CI 0.98–0.99; *p* < 0.001). Every one minute increase in the actual waiting time is associated with 2% decrease in the chance of the patient perceiving the wait as ‘very short’. In other words, after 30 min of waiting, patients’ perceived waiting time as ‘very short’ would decline by 81%.

## Discussion

Our results showed that 41% of respondents expressed agreement on satisfaction in the waiting room and 30% strongly agreed. Mean waiting time expected by the patient was 94.85 min. Patients’ estimation of the mean reasonable waiting time before seeing a doctor was 65.42 min, with a mean of 78 min for the actual waiting time. Twenty-four percent of respondents described their perception of the waiting time as being short, 43.5% as acceptable, and 32.4% as long. Indeed, the average waiting time was considered as very short, short and acceptable until a mean of 63 min. Interestingly, 53.2% reported that they did not receive information from caregivers about the waiting time. The model used for this study (annex 1) identified four significant predictors of the wait perception in decreasing order: appropriate assessment of emergency level by caregivers; feeling of being forgotten; respect of privacy; and the exact waiting time. Of note, 23% (115/499) of respondents reported to have felt forgotten. Regarding the appropriate assessment of emergency level by caregivers, only 4.5% said that they disagreed (‘strongly disagree’ and ‘disagree’). Similarly, 8.2% disagreed with the organization of the emergency unit; 67.5% were interested to wait elsewhere, and 84.3% said that their privacy was respected (‘agree’ and ‘strongly agree’).

### Appropriate assessment of emergency level by caregivers

Accurate assessment of the emergency level by caregivers is the strongest influencer of the wait perception. Reasons for attending EDs are linked, but not only to the perception of situation urgency. Some patients in Nederland, Australia also mentioned that they went to the ED to be able to see a doctor and have any tests or X-rays all done in the same place or because they did not want to wait for an appointment with their general practitioner [31, 32]. Furthermore, nurses and doctors may disagree about the patient’s triage category [33]. The discrepancy in severity assessment between caregivers and patients has also been highlighted in the research of Toloo et al. [17]. In their study, they reported that almost 50% of patients had the feeling of being undertriaged and 20% expected a lower priority than the actual triage category. The correlation between the perceived priority and actual triage category was weak. In a patient-centred approach, caregivers should be encouraged to explain clearly to patients the reasons for their classification at a given level and why other patients may be possibly seen before them by medical staff. Therefore, it would be important to create different support materials that allow the patient to understand the reasons for his/her assigned triage category. In addition, more consideration should perhaps be given to the perceived urgency by patients as Toloo et al. [17] showed that this is associated with an expected higher priority triage.

### Feeling of having been forgotten

The feeling of having been forgotten is identified as the second strongest influencing factor of the wait perception. Although this criteria was not retrieved in our literature review on the topic, we found one article in a related domain by Gilmartin et al. [34] who reported that patients felt abandoned during preoperative wait in one care centre. In this study, the main reasons of feeling abandoned was the lack of information about the delay, the process, and poor interaction with caregivers. In our setting, when a patient is seated in the waiting room, s/he usually has to wait until a nurse comes to take him/her to an examination room. During this entire time, the patient is isolated and often lacks information and is therefore unsure whether s/he has been forgotten by staff.

### Link between the wait perception and actual time

The statistical analysis showed that the actual waiting time influences significantly its perception and that patients are ready to wait up to 1 h on average before considering the wait as excessive. This finding is supported by Sanober et al. [35] showing that patients were willing to wait up to 2 h before leaving the ED without being seen. Another study [36] demonstrated that patients felt that they should be seen within 1 h on average, but expected to wait 2.1 h. After 2 h, people wanted to leave the ED before seeing a medical doctor.

Eighty percent of patients visiting our emergency unit were classified as level 3 and must be seen by a doctor within a delay of 120 min according to the Swiss Emergency Triage Scale. Our observations highlighted that we must either reduce this waiting time or act on it by changing the wait perception. For example, it is known that the perception of waiting time, efficiency and the clinical skills of the emergency doctor is improved with periodic personal interaction and the provision of clinically-based information [37].

### Link between the confirmation and perceived time

As described by Thompson et al. [9, 13], we showed that the wait perception was correlated with the discrepancy between the reality and the expectation of the wait. A gap between performance and expectations generates a ‘disconfirmation’. Moreover, it is known that if a patient expects to wait longer than the actual wait, s/he is more satisfied, independent of the length of the waiting time [5]. Antinides et al. [10] showed that waiting time ‘fillers’, such as repeated information about wait duration, length of the queue, and music positively influence the time perception. This should also encourage caregivers to provide an overestimation of waiting time. This mechanism has been observed in other areas, such as waiting for transportation, where providing real-time information has demonstrated a positive influence on time perception. In our emergency unit, nurses give a personal estimate of the waiting time to at least 36% of patients.

### Respect of privacy

Respect of privacy was the least significant predictor. The patients in our units wait in a semi-closed room with a television, bottles of water and a choice of different newspapers. Nurses come regularly to the waiting room to check the pain scale and sometimes provide information on an estimate of waiting time. The design of the waiting environment can be a significant factor in improving patient satisfaction. In a questionnaire on patients’ privacy and satisfaction in an urban university-based hospital ED [38], 75% agreed and strongly agreed that privacy was very important for their emergency care. Following these results, an intervention including redesigning the ED environment, process management and staff education was implemented and showed significant improvements with patient perceptions on increased privacy and satisfaction. In our study, 84% of patients agreed and strongly agreed that their privacy was respected. Questions regarding discomfort showed that most patients (> 60%) strongly disagreed/disagreed and considered that their comfort was disrupted by other patients, by the coming and going of caregivers, or by the lack of distractions.

### Study limitations

Our study has some limitations. The questionnaire was developed specifically for this study as no existing questionnaire in the literature allowed us to evaluate the perception of waiting time. As the questionnaire validity and reliability have not been tested, our results cannot be generalized without additional evaluation. The questionnaire was only available in French and excluded patients included those who were in too much pain, unable to read French, with ocular problems or who did not want to complete it. It is also possible that the nurses did not give the questionnaire during busy shifts. In addition, as the questionnaires were distributed by nurses, the social desirability effect could have had an impact on the patient’s decision to participate and on their responses [39]. Another limitation is that the analysis was only in one unit of our ED. No response rate was calculated. Due to financial constraints, the questionnaire could only be distributed during a three-month period and this did not allow to reach the 700 participants targeted with the sample size calculation based on the rule of event per variable of 50. However, we did reach the minimal recommended sample size of 500 participants when using logistic regression for observational studies [40]. Of note, our evaluation took place at the end of the waiting process and did not take into account the quality of care dispensed. As this can influence overall patient satisfaction at the end of the entire process, it can be considered as a limitation.

## Conclusion

In emergency care, medical consultations are not scheduled and the patient must almost always wait, especially non-urgent cases. Overcrowding of EDs is a major problem worldwide, with a negative impact on the quality of care. In the throughput process, the waiting time until the medical contact impacts on the overall patient satisfaction. Our study revealed that during the wait until the medical encounter, there is a ‘golden hour’ when the patient is willing to wait and a perceived waiting time of under 60 min is acceptable. This is consistent with the results of studies showing that patients may leave the ED after 2 h without seeing a doctor. Therefore, it can be recommended to provide information to patients if they have been waiting for more than 1 h.

The two strongest predictors of the perceived waiting time before seeing a doctor were appropriate assessment of the emergency level by caregivers and the feeling of being forgotten. Thus, ED staff should be particularly attentive to act on these factors. A solution could be to perform a more patient-centred triage, with more basic information and different support materials to allow the patients to understand the process according to their sociocultural background. Further research is needed to understand the psychological profile of patients who over- or underrate their urgency, as well as a more detailed analysis to discover the needs of patients who are waiting before the first medical contact.

Some of our results revealed avenues for improvement. For example, 47.9% of patients were unable to situate themselves in the waiting queue and 67.5% reported an interest to wait outside the waiting room (cafeteria, at home, somewhere else). These facts informed us that we should focus on interventions to improve the perceived waiting time and reduce the feeling of being forgotten. Consideration could be given also to moving the wait: the patient could wait elsewhere or be seen rapidly by a doctor and then wait (move the wait after the first medical contact). Furthermore, it could be interesting to retest the wait perception at the time of patient discharge as it would permit a comparison with the waiting perception at the beginning of the process before seeing a doctor and help to monitor the impact of any interventions.

## Data Availability

Supporting data can be accessed at the Springer Nature repository.

## Abbreviations

CIS: Clinical information system
ED: Emergency department
SET: Swiss Emergency Triage Scale
